# Blended Therapy From the Perspective of Mental Health Professionals in Routine Mental Health Care: Mixed Methods Analysis of Cross-Sectional Survey Data

**DOI:** 10.2196/78079

**Published:** 2026-01-06

**Authors:** Annalena Maria Kneubühler, Elianne von Känel, Kristina Grgic, Ena Munkovic, Thomas Berger, Laura Luisa Bielinski

**Affiliations:** 1 Department of Clinical Psychology and Psychotherapy, Institute of Psychology, University of Bern, Fabrikstrasse 8, Bern, 3012, Switzerland, 41 31 684 39 37

**Keywords:** blended therapy, digital interventions, implementation, routine care, therapist attitudes

## Abstract

**Background:**

Digital interventions play an innovative role in the treatment of mental health disorders, offering evidence-based solutions across a wide range of conditions. Blended therapy (BT), which integrates digitally delivered interventions with face-to-face therapy, has shown promise. However, challenges such as low uptake hinder widespread implementation. Mental health professionals are key stakeholders for the adoption of BT in routine care settings.

**Objective:**

This study explores mental health professionals’ perspectives on BT, specifically assessing their perceived knowledge of, acceptance of, usage of, and perceptions of different BT types. Additionally, it examines mental health professionals’ perceived advantages and disadvantages of BT, challenges associated with implementation, and wishes toward the future application of BT.

**Methods:**

A survey study was conducted among 203 mental health professionals (152 psychological psychotherapists and 51 psychiatrists, including also individuals in training) in Switzerland. The data were analyzed using both quantitative methods and qualitative content analysis.

**Results:**

Participants reported limited knowledge of BT (mean 2.71, SD 1.32), attitudes toward BT were somewhat positive (mean 5.25, SD 1.34), and acceptance was moderate (mean 3.64, SD 1.20). Among various digitally delivered interventions, teletherapy (video) was most frequently integrated with face-to-face treatment and considered more suitable for BT than chat, email, or new technologies. More than 75% (n=152) of the respondents deemed BT appropriate for the treatment of affective (mood) disorders (F30-F39) and for the treatment of neurotic, stress-related, and somatoform disorders (F40-F48; ICD-10). The qualitative analyses of open-ended questions highlighted key advantages of BT as perceived by mental health professionals. These include increased treatment flexibility, the ability to outsource therapy components, and enhanced treatment efficiency. However, disadvantages such as increased effort and potential disruptions to the therapeutic relationship were also noted. Participants identified barriers to BT implementation, including financing and data security concerns. To facilitate BT adoption, respondents emphasized the desire for better cost coverage, easy access to digitally delivered interventions, and seamless integration of digital tools into face-to-face therapy.

**Conclusions:**

The findings indicate that mental health professionals report limited knowledge of BT and consider it more suitable for certain disorders than others. Moreover, from their perspective, while BT offers advantages, it also presents disadvantages. Addressing mental health professional knowledge gaps, alongside resolving perceived implementation barriers, may be key to the successful future implementation of BT in routine mental health settings.

## Introduction

### Digital Interventions to Treat Mental Health Disorders

The evidence base supporting the efficacy of digital psychological interventions to treat mental health conditions and problems is extensive and continues to grow [1-5]. These interventions encompass a broad spectrum, ranging from fully self-guided programs [4], designed to, for example, provide support for individuals who might otherwise lack access to traditional therapy, to more integrated approaches that combine digital elements with face-to-face therapy [6,7].

### Blended Therapy

Blended therapy (BT) in the mental health context refers to the combination of a digitally delivered intervention and face-to-face therapy [8,9]. Digital interventions and face-to-face therapy can be combined in different ways, ranging from digitally delivered interventions provided prior to or as aftercare to face-to-face therapy to interventions interwoven during a course of face-to-face therapy. The first systematic review on BT [9] describes the potential of this type of treatment regarding both study dropout and time savings in therapy. A more recent systematic review and meta-analysis [2] describes the feasibility of BT and reports on BT effects. BT interventions were more effective or noninferior to treatment as usual (defined as pharmacological or psychological intervention and standard medical care), with a moderate-to-large effect size in the treatment of depression (Cohen *d*=–1.1, 95% CI –0.6 to –1.6; *P*<.001). For anxiety outcomes, the meta-analysis reported a small, nonsignificant effect size (Cohen *d*=–0.1, 95% CI –0.3 to 0.05; *P*=.17). The findings also highlight higher effect sizes for blended interventions with supplementary design, fewer (≤6) face-to-face sessions, and a lower ratio (≤50%) of face-to-face versus digital sessions [2].

### BT in Routine Mental Health Care Settings

Various studies highlight the successful integration of digital interventions with face-to-face therapy in routine mental health care settings. Reported benefits include enhanced efficacy and effectiveness [6,10]. Another study reported no significant difference in symptom change over time between the blended and control group [11]. Moreover, research also underscores challenges and limitations associated with BT. For instance, a recent large-scale study conducted in routine care settings in Germany by Schaeuffele et al [12] identified issues such as adherence as hindering factors for implementation.

### The Perception of Health Care Providers

Mental health care providers play a pivotal role in the successful implementation of BT. Their attitudes can influence practical application [13,14]. While lagging implementation of digitally delivered interventions appears to be a recurring trend across multiple European countries [14,15], the COVID-19 pandemic has led to greater uptake and acceptance [16,17]. BT has been perceived more favorably than stand-alone digitally delivered interventions by clinicians [13,18,19]. However, reservations toward BT among mental health professionals have also been reported. For example, concerns regarding the therapeutic alliance, patient engagement, data security, the therapeutic process, and work-life balance [20,21] may impact providers’ willingness to adopt BT.

### Aims

Although the evidence base on BT is growing, several research gaps remain. Most existing studies have focused on feasibility and clinical outcomes, while less is known about how BT is perceived and implemented in routine mental health care settings. Detailed insights into health professionals’ perceived knowledge, attitudes, and acceptance of BT in Switzerland are limited, and both qualitative and quantitative analyses are required to adequately examine these specific topics. This study reports on a mixed methods analysis using data from a survey completed by mental health professionals and mental health professionals in training in Switzerland. Specifically, the study explores the following research questions: (1) What is the current level of perceived knowledge, attitude toward, and acceptance of BT among psychological psychotherapists and psychiatrists (including those in training)? (2) How is BT currently used by participants? (3) How do mental health professionals perceive the suitability of different digitally delivered interventions for BT purposes, and which types of BT are they willing to use in the future? (4) What are the perceived advantages and disadvantages of BT, what challenges are there regarding implementation and what are mental health professionals’ wishes for the future regarding BT?

## Methods

### Study Design

This study examined BT from the perspective of psychological psychotherapists and psychiatrists (also those in training) in Switzerland, using a cross-sectional, open online-survey approach. Participants filled out the survey between October 2023 and February 2024.

### Ethical Considerations

The study received approval from the Ethics Commission of the Faculty of Human Sciences, University of Bern (ID: 2023-09-04). Participants received no incentive or compensation for participation. All participants provided informed consent to participate. The survey was conducted with no collection of direct identifiers such as names, contact information, IP addresses, or geographic location. The survey included limited demographic variables (eg, gender and job category) for analytical purposes. Any potentially identifying information contained in free-text responses was removed or generalized prior to analysis.

### Measures

A total of 23 survey questions from a comprehensive survey on the topic of BT were used to answer the research questions presented in this study. The full survey translated from German to English can be found in Multimedia Appendix 1 along with the instructions participants received. Survey questions were built on previous literature [8,14,18-20,22,23]. The survey was provided through Qualtrics [24] and was tested prior to dissemination with several test-runs by the authors of this study. Users’ IP addresses were not recorded. The survey was available in German and French for participants. Each survey page included a back button.

To answer research question 1, we assessed mental health professionals’ perceived knowledge of, general attitude toward, and acceptance of BT. Acceptance of BT was operationalized following Braun et al [22] using 3 specific items: “I could imagine including BT into my work”; “I intend to try out BT in my work within the next year”; “How high is your intention to use BT in your work ever?”. The first 2 questions were assessed on a 5-point Likert scale ranging from 1 (totally disagree) to 5 (totally agree). The third item was rated on a 0-to-100 scale and converted into a 5-point Likert scale to measure the strength of intention. A mean value was calculated from all 3 items to quantify the acceptance of BT. Based on prior research [22], the mean acceptance score was categorized as low (1‐2.34), moderate (2.35‐3.67), or high (3.68‐5). To answer research question 2, we assessed both past use of BT and current use of the different digital intervention modalities for BT (eg, teletherapy [video], chat, email, self-management, new technologies). To answer research question 3, the perceived suitability of different digitally delivered interventions (teletherapy [video], chat, email, self-management interventions, and new technologies) for BT was assessed. The suitability of BT for different *ICD-10* (*International Statistical Classification of Diseases, Tenth Revision*) [25] disorders was also assessed. Moreover, the future willingness to use digital interventions in relation to various points of treatment and in different settings (outpatient, day clinic, inpatient, acute inpatient) was assessed. To answer research question 4, participant answers to 4 open-ended questions were examined. Detailed item wording and the precise response scales for all items used to answer the research questions are reported in Multimedia Appendix 2.

### Statistical Analyses

All quantitative analyses were conducted using SPSS (version 29; IBM Corp). Descriptive statistics (means, SDs, frequencies, and percentages) were used to address the primary research questions. Inferential statistics were applied to explore patterns and group differences. Repeated-measures ANOVAs were conducted where appropriate, with Greenhouse-Geisser corrections applied when the assumptions of sphericity were violated. For participants with missing values, listwise deletion was applied. Effect sizes *ηp*² were reported to aid interpretation for ANOVAs. Pairwise comparisons were Bonferroni corrected and Cohen *dz* was reported as effect size. For dichotomous outcomes, Cochran *Q* and follow-up McNemar tests with Bonferroni corrections were used, and Cohen *g* was reported as effect size for the pairwise comparisons. For group comparisons between professional groups and between those in training versus not in training, independent sample *t* tests were conducted. All significance tests were 2-sided with a significance level of *α*=.05.

The perceived advantages and disadvantages of BT, as well as implementation challenges and future wishes, were analyzed using an inductive content analysis approach as outlined by Mayring [26]. This approach is well suited to qualitative analyses that stay close to the semantic content of responses and allow for integration of qualitative and quantitative elements, such as reporting category frequencies. Separate inductive analyses were conducted for each area (advantages, disadvantages, challenges, and future wishes). Following Mayring’s [26] category formation steps, KG coded all responses, assigning codes to the raw material. Multiple codes could be assigned per survey item response, but the same code could not be assigned twice. In the next step, categories and subcategories were discussed collaboratively with LLB, and the category system was refined in an iterative process of repeated reviewing of the material and adjusting of categories. Finally, KG coded the entire material set with the final categories and subcategories that were formed. All analyses were conducted using Microsoft Excel (version 2016). Anchor examples for the categories were taken verbatim from participant answers.

Due to variation in response rates across survey items, sample sizes are reported throughout the study. Detailed information on item-level missingness is provided in Multimedia Appendix 3. No weighting of items or propensity scores was used to adjust for the nonrepresentative sample.

## Results

### Recruitment  

**Figure 1.**
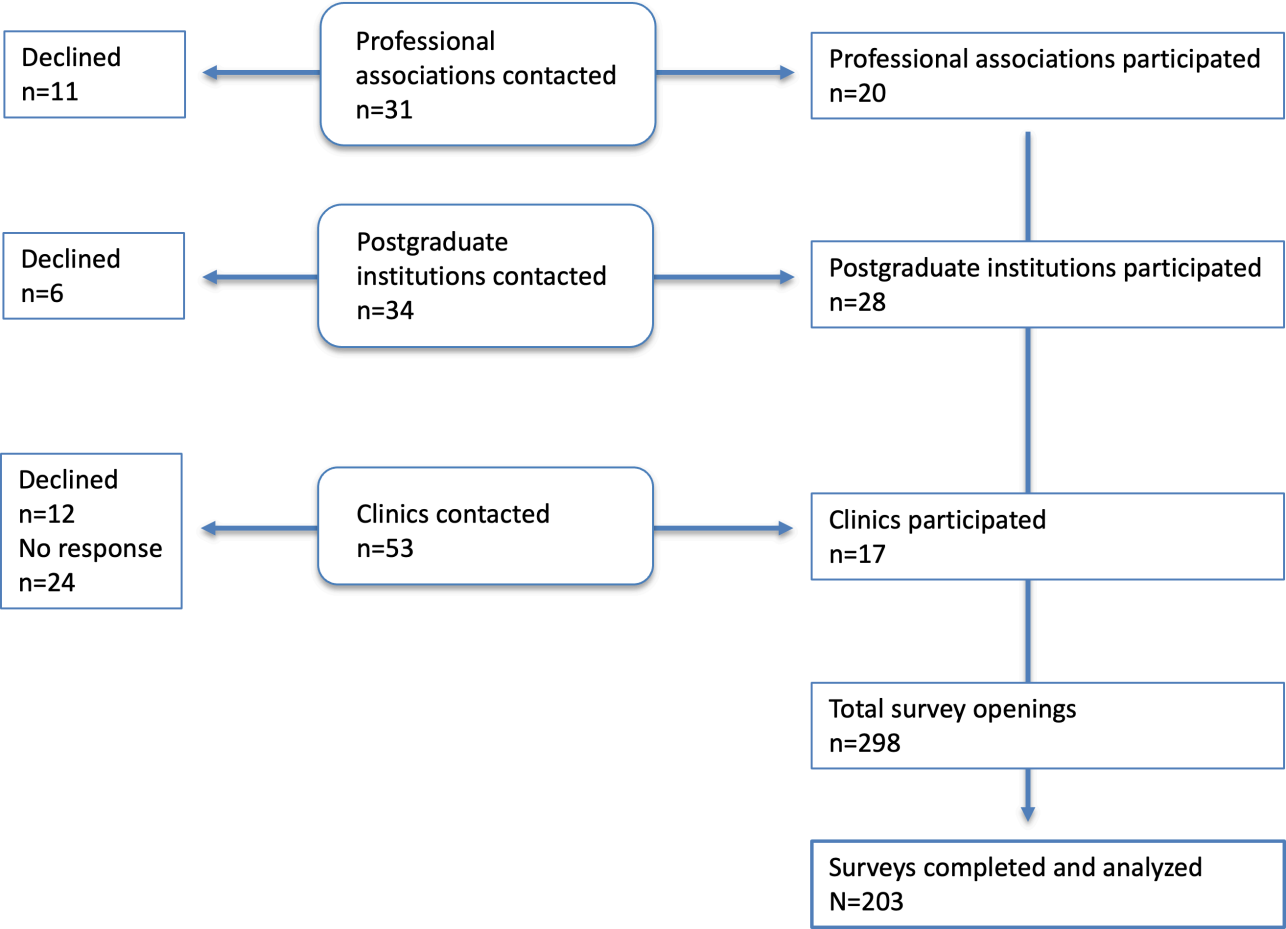
Flow chart depicting recruitment pathways for the survey.

To recruit participants, professional associations, psychotherapy training institutes, and psychiatric clinics across Switzerland were contacted and invited to disseminate the study link to their members or personnel via internal communication channels. Up to 3 reminder emails were sent to each organization. The contacted clinics were identified from a public registry provided by the Schweizerisches Institut für ärztliche Weiter- und Fortbildung [27]. Overall, a broad range of professional and institutional stakeholders were approached, of whom a subset actively declined participation due to staff shortages, an overload of inquiries, or other individual reasons. A detailed overview of the recruitment process, including the number of institutions contacted and participating, is presented in Figure 1. The survey was opened 298 times, and the 203 responses that reached the end of the survey were included in the analysis.

### Sample

An overview of sample characteristics provided for the 203 survey completions (visited each survey page until the end) is presented in Table 1.

**Table 1.** Sample characteristics.

Sample characteristic	Values
Gender, n (%)	
Man	61 (30.0)
Woman	141 (69.5)
Nonbinary (diverse)	1 (0.5)
Age (y), mean (SD; Min^a^, Max^b^)	45.9 (14.1; 24, 79)
Professional group, n (%)	
In training to become a federally recognized psychotherapist	61 (30.0)
Federally recognized psychotherapist	91 (44.8)
Specialist in psychiatry and psychotherapy	41 (20.2)
In training to become a specialist in psychiatry and psychotherapy	7 (3.4)
Specialist in child and adolescent psychiatry and psychotherapy	3 (1.5)
Years of training, n (%)^c^	
First year	17 (25.0)
Second year	19 (27.9)
Third year	11 (16.2)
Fourth year	12 (17.6)
Fifth year	6 (8.8)
Sixth year	1 (1.5)
>6	2 (2.9)
Work experience in psychotherapeutic practice (y), n (%)	
None	1 (0.5)
<1	12 (5.9)
1‐5	62 (30.5)
6‐10	22 (10.8)
11‐15	32 (15.8)
>15	74 (36.5)
Therapeutic orientation, n (%)^d^	
Cognitive-behavioral therapy (cognitive or cognitive-behavioral approach)	112 (55.2)
Depth-psychological or psychodynamic	37 (18.2)
Psychoanalytic	32 (15.8)
Systemic	63 (31.0)
Humanistic	48 (23.6)
Other	44 (21.7)
Current work setting, n (%)	
Outpatient	144 (70.9)
Partial inpatient or day clinic	3 (1.5)
Inpatient	22 (10.8)
Mixed (outpatient and inpatient)	19 (9.4)
Mixed (outpatient and partial inpatient)	6 (3.0)
Mixed (partial inpatient and inpatient)	6 (3.0)
Currently not employed	3 (1.5)

a Min: minimum.

b Max: maximum.

c This applies to the subgroups in training to become a federally recognized psychotherapist and in training to become a specialist for psychiatry and psychotherapy.

d Multiple responses were possible.

### Perceived Knowledge of, Attitude Toward, and Acceptance of BT

The overall sample reported a mean (SD) of 2.71 (1.32) for perceived knowledge, corresponding to a value of 3 (“a little”). A total of 44 (21.7%) participants reported having no knowledge of BT, and only 4 (2.0%) participants reported having a great deal of knowledge of BT. See Table S1 in Multimedia Appendix 4 for full descriptive data. Regarding attitude toward BT, the overall sample reported a mean (SD) of 5.25 (1.34), corresponding to a value of 5 (“somewhat positive”). For BT acceptance, the mean (SD) was 3.64 (1.20), corresponding to moderate acceptance [22]. Analyses of differences in knowledge of BT, attitude toward BT, and acceptance between professional groups and between those in training versus those not in training are provided in Tables S2-S4 in Multimedia Appendix 4.

### Use of BT

**Figure 2.**
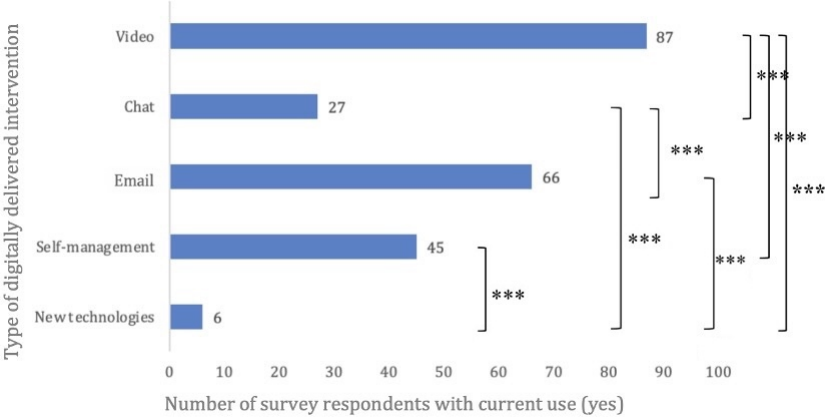
Current use of different digitally delivered interventions in combination with face-to-face therapy. N=203. ***P<.001. The number of participants who answered *yes* to current use of different digitally delivered interventions in combination with face-to-face therapy by intervention type is displayed. Multiple digitally delivered intervention uses were possible per participant.

Of the total sample, 125 (61.6%) participants reported having used some form of BT in the past. The mean score for current use across the sample was mean (SD) of 2.14 (1.22), corresponding to a scale value of 2 (“rarely”). Figure 2 shows the number of participants who answered “yes” to currently using different types of digitally delivered interventions as part of therapy. A Cochran *Q* test indicated significant differences in current use across intervention types (Q₄=136.58; N=203; *P*<.001). Pairwise McNemar tests were conducted to further examine these differences. To control for type I error inflation due to multiple comparisons (*k*=10), a Bonferroni correction was applied. After Bonferroni correction, all differences between digitally delivered intervention formats remained significant except for chat versus self-management (*P*=.21), self-management versus email (*P*=.12), and email versus video (*P*=.09). See Table S5 in Multimedia Appendix 4 for a full overview of the pairwise comparisons.

Comparisons between professional groups and those in training versus not in training regarding current use of BT are also shown in Tables S6 and 7a and 7b in Multimedia Appendix 4.

### Perceived Suitability of Different Digitally Delivered Interventions for BT

**Figure 3.**
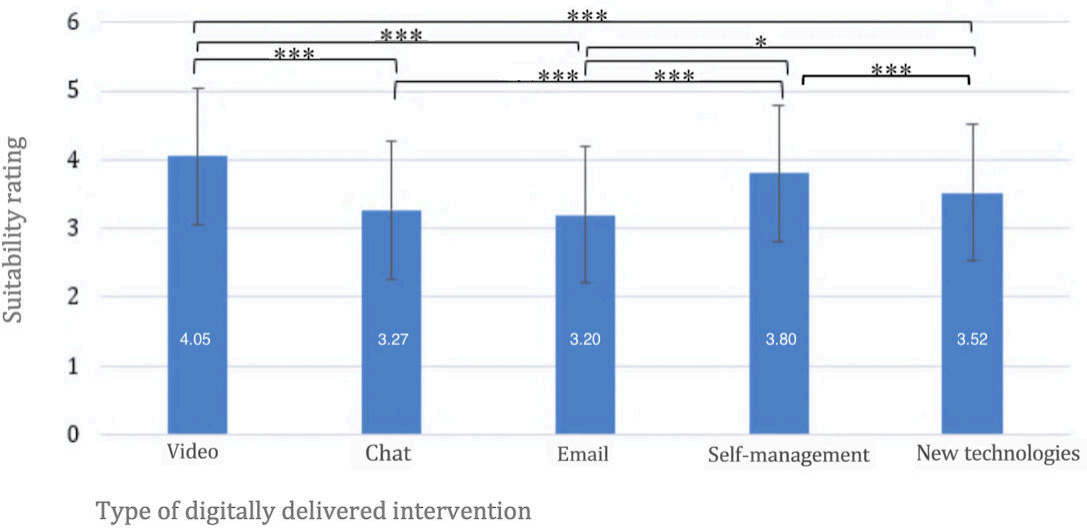
Suitability ratings of different digitally delivered interventions for blended therapy (BT). N=203. *P<.05; **P<.01; ***P<.001. Means are displayed in white. Error bars represent ±1 SD. Axis extends beyond the maximum response option to display full error bars; no respondent values exceeded the upper scale limit (5).

Participants rated the suitability of different digitally delivered intervention types for BT. A repeated-measures ANOVA showed significant differences in suitability ratings between the intervention types (*F*_3.48, 702.27_=30.57; *P*<.001; *ηp*²=0.13). Video conferencing was rated as significantly more suitable than interventions via chat (mean difference [MD]=0.78; *P*<.001; *dz*=0.57), email (MD=0.86; *P*<.001; *dz*=0.60), and new technologies (MD=0.54; *P*<.001; *dz*=0.43). Chat was rated significantly less suitable than self-management interventions (MD=−0.53; *P*<.001; *dz*=0.39). Email interventions were rated significantly less suitable than self-management interventions (MD=−0.60; *P*<.001; *dz*=0.47) and new technologies (MD=−0.32; *P*=.01; *dz*=0.23); see also Figure 3. Table S8 in Multimedia Appendix 4 shows an overview for full descriptive data.

### Suitability According to *ICD-10* Mental Health Disorder Categories * *

**Figure 4.**
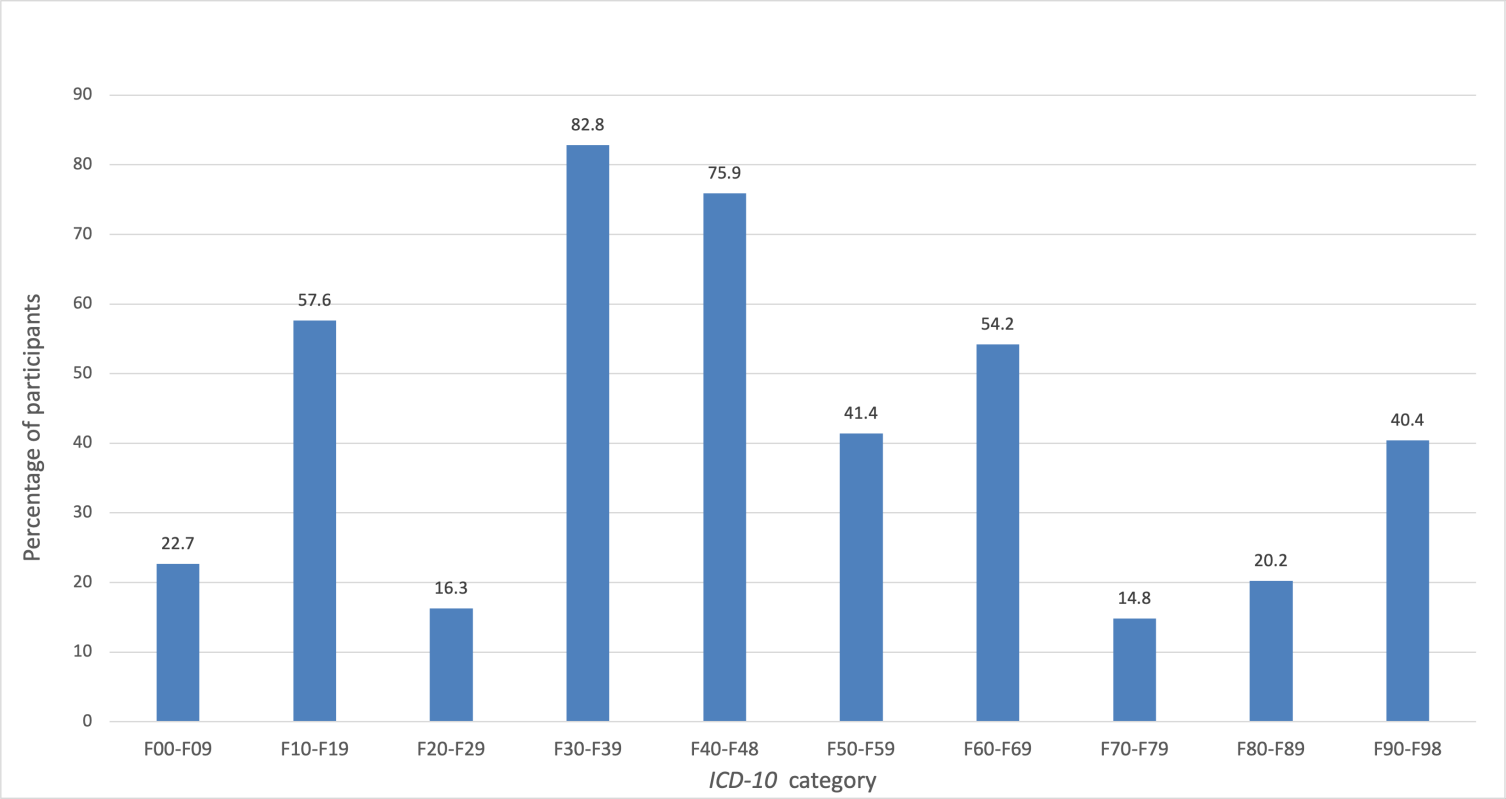
Descriptive data on participants who rated blended therapy (BT) as suitable for different mental health disorders. N=203. Percentage of participants who said BT was suitable is displayed for each disorder group. F00-F09=Organic, including symptomatic, mental disorders; F10-F19=Mental and behavioral disorders due to psychoactive substance use; F20-F29=Schizophrenia, schizotypal, and delusional disorders; F30-F39=Mood [affective] disorders; F40-F48=Neurotic, stress-related, and somatoform disorders; F50-F59=Behavioral syndromes associated with physiological disturbances and physical factors; F60-F69=Disorders of adult personality and behavior; F70-F79=Intellectual disabilities; F80-F89=Disorders of psychological development; F90-F98=Behavioral and emotional disorders with onset usually occurring in childhood and adolescence. *ICD-10*: *International Statistical Classification of Diseases, Tenth Revision*.

As shown in Figure 4, over 75% (n=152) of the participants considered BT suitable for treating Mood disorders (F30-F39) and Neurotic, stress-related, and somatoform disorders (F40-F48). In contrast, very few participants endorsed BT as suitable for Schizophrenia and delusional disorders (F20-F29) or Intellectual disabilities (F70-F79). The Cochran *Q* test indicated significant differences in suitability across disorder categories (*Q*(9)=558.55; *P*<.001). Pairwise McNemar tests with Bonferroni correction further explored these differences (see Table S9 in Multimedia Appendix 4).

### Willingness to Use at Different Points During Treatment

**Figure 5.**
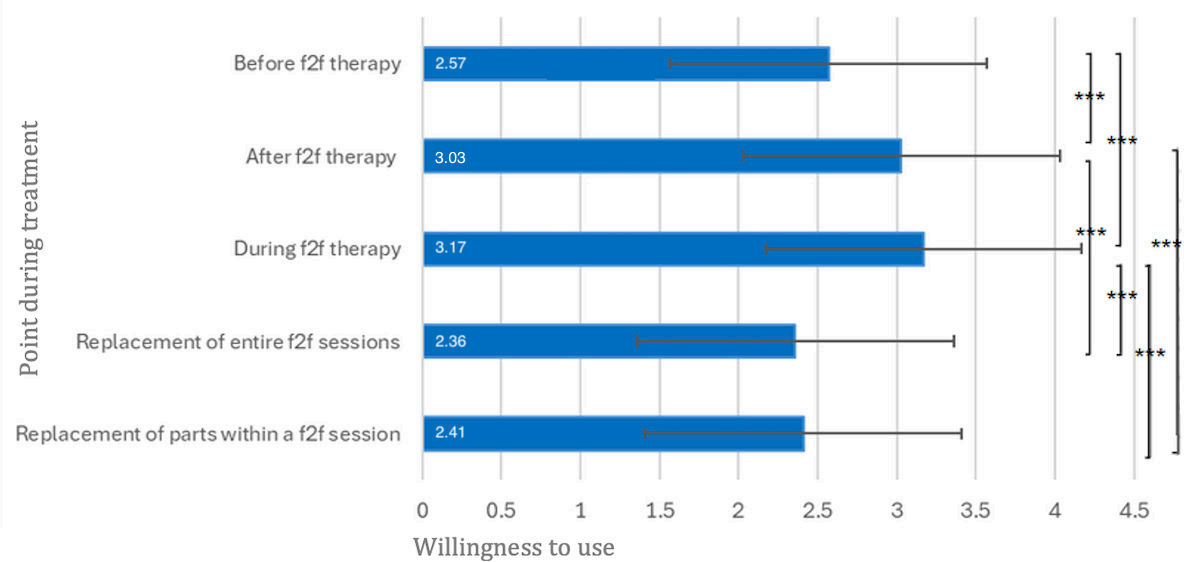
Future willingness to use digitally delivered interventions at different points during treatment. N=191. ***P<.001. Definitely no=1, Rather no=2, Rather yes=3, and Definitely yes=4. Means are displayed in white. Error bars represent ±1 SD. Axis extends beyond the maximum response option to display full error bars; no values exceeded the upper scale limit (4). f2f: face to face.

The future willingness to use digitally delivered interventions in relation to various points of treatment is illustrated in Figure 5. The mean (SD) values were 2.57 (1.06) for use before psychotherapy, 3.03 (0.89) for after psychotherapy, and 3.17 (0.84) for during psychotherapy. These values each correspond to scale point 3 (“rather yes”). For use as a substitute for individual sessions, the mean (SD) was 2.36 (1.12), and for use as a substitute for individual parts of a session, the mean (SD) was 2.41 (1.03), which in both cases corresponds to scale point 2 (“rather no”).

A repeated-measures ANOVA indicated significant differences between the application points (*F*_3.11, 591.20_=41.34; *P*<.001; *ηp*²=0.18). Willingness to use digitally delivered interventions was significantly lower before psychotherapy than after (MD=−0.46; *P*<.001; *dz*=0.51) and during psychotherapy (MD=−0.60; *P*<.001; *dz*=0.54). Willingness to use digitally delivered interventions after psychotherapy was significantly higher than for the replacement of individual sessions (MD=0.67; *P*<.001; *dz*=0.51) and for the replacement of individual parts of sessions (MD=0.62; *P*<.001; *dz*=0.63). Similarly, willingness to use digitally delivered interventions during psychotherapy was significantly higher than for the replacement of individual sessions (MD=0.81; *P*<.001; *dz*=0.68) and for the replacement of individual parts of sessions (MD=0.76; *P*<.001; *dz*=0.75). No other pairwise differences were statistically significant (see Figure 5).

### Willingness to Use Across Different Settings  

**Figure 6.**
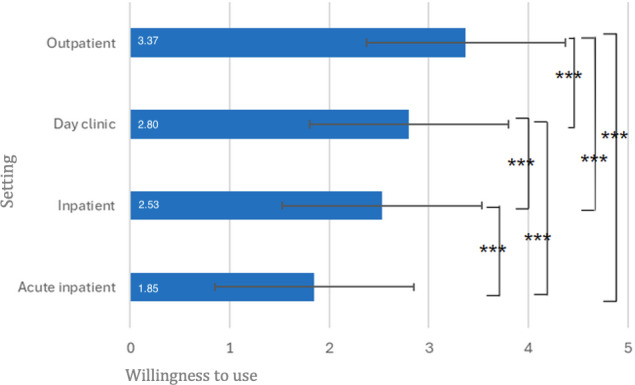
Future willingness to use blended therapy (BT) in different settings. N=172. ***P<.001. Definitely no=1, Rather no=2, Rather yes=3, and Definitely yes=4. Means are displayed in white. Error bars represent ±1 SD. Axis extends beyond the maximum response option to display full error bars; no values exceeded the upper scale limit

The future willingness to use digitally delivered interventions varied across treatment settings (Figure 6). The mean (SD) ratings were 3.37 (0.72) for outpatient settings, 2.53 (0.88) for inpatient settings, 2.80 (0.89) for day clinic settings, and 1.85 (0.82) for acute inpatient settings. A repeated-measures ANOVA showed significant differences in willingness to use between the settings (*F*_2.75, 471.00_=185.79; *P*<.001; *ηp*²=0.52). Willingness to use was significantly lower in the acute inpatient setting than in the inpatient (MD=−0.68; *P*<.001; *dz*=0.84), day clinic (MD=−0.94; *P*<.001; *dz*=1.06), and outpatient (MD=−1.52; *P*<.001; *dz*=1.53) settings. It was also significantly lower for inpatient than day clinic (MD=−0.26; *P*<.001; *dz*=0.36) and outpatient (MD=−0.84; *P*<.001; *dz*=0.95) settings and significantly lower for day clinic than outpatient settings (MD=−0.58; *P*<.001; *dz*=0.71; see Figure 6).

### Advantages and Disadvantages of BT

A total of 148 participants reported on the advantages of BT (Table 2). A total of 233 codes were coded. These were grouped into 4 main categories and 14 subcategories. At least 141 participants reported on the disadvantages of BT (Table 3). A total of 215 codes were generated, which were grouped into 5 main categories and 23 subcategories.

**Table 2.** Advantages of blended therapy (BT).

Category	Subcategory	n^a^	Anchor example
Therapy-related factors	Flexibility	79	Flexibly being able to cater to individuality of patients with therapy (Survey 5)
	Outsourcing therapy elements	43	Delegation of in-depth psychoeducation (Survey 101)
	Efficiency	35	More effective and thus shorter therapy duration (Survey 26)
	For different points in treatment	10	Bridge wait times (Survey 141)
	Problem-specific or transdiagnostic benefits	8	Autism/Asperger (Survey 37)
Relationship factors	Strengthening the therapeutic relationship	13	Strengthening of commitment and compliance in therapy (Survey 129)
Patient factors	Increase in self-efficacy	12	Increasing self-efficacy of patients (Survey 29)
	Lowering barriers	6	Reducing barriers to do with fear (Survey 17)
	Access to topics	5	Access to topics that patients can’t talk about in face-to-face sessions (Survey 81)
	Increase in therapy motivation	4	Change in therapy motivation is measurable (Survey 129)
	Attractive option for therapy	2	Making therapy more attractive (Survey 37)
Mental health professional factors	Capacity	9	More clients (Survey 66)
	Location-independent work	4	Work in home office (Survey 26)
	Relief	3	Relief (Survey 97)

a n: number of participants with code.

**Table 3.** Disadvantages of blended therapy (BT).

Category	Subcategory	n^a^	Anchor example
Technological, organizational, and legal aspects	Technical prerequisites	15	Technology needs to work (Survey 170)
	Billing	11	Unclear billing options (Survey 31)
	Costs	10	Investment in further training and procurement (Survey 82)
	Data security	12	Need to engage with data security topic (Survey 6)
Practical implementation of interventions	Effort	24	Additional effort to gather information and review suitable options (Survey 40)
	Limitation of holistic treatment	15	The therapeutic vessel may become watered down (Survey 139)
	Not suitable for some interventions	9	Behavioral observation becomes more difficult (Survey 45)
	Progress monitoring	5	Less control over the development of the patients’ condition (Survey 137)
	Competition among offers	1	Competition between individual offerings (Survey 33)
Interpersonal interaction	Therapeutic relationship	21	The relationship is interrupted (Survey 37)
	Reduced contact	11	Reduction of human contact (Survey 65)
	Nonverbal communication	3	Lack of nonverbal communication (Survey 132)
Patient-related challenges	Indication	24	My patients with psychosis will often not use it (Survey 145)
	Avoidance behavior	13	Enables avoidance of interactions with others (Survey 37)
	Lack of motivation	4	Patients’ motivation is rather unclear and uncertain (Survey 61)
	Overwhelm	3	Overwhelm of the patient (Survey 65)
	Loss of autonomy	2	Misunderstandings in autonomous work (Survey 187)
	Media consumption	1	Dependency on tools (Survey 65)
Personal and professional challenges	Lack of knowledge	10	The topic is unclear to me: too little experience (Survey 106)
	Accessibility	8	Having to organize when one is not reachable, etc (Survey 96)
	Cognitive strain Professional field	4 4	Increased tiredness for therapist when contact is not face-to-face (Survey 96) Loss of individuality and spontaneity in individual cases, emergence of boredom even for the therapist (Survey 173)

a n: number of participants with code.

### Challenges for Implementation and Wishes for the Future

A total of 129 individuals were included in the qualitative analysis of open-ended responses to the question on challenges regarding BT implementation. A total of 7 main categories and 15 subcategories were identified. At the subcategory level, a total of 206 codes were assigned, as shown in Table 4. Additionally, 108 individuals were included in the analysis of the open-ended question on wishes for the future regarding BT. A total of 151 codes were generated and grouped into 15 categories, which are presented in Table 5.

**Table 4.** Challenges regarding implementation.

Category	Subcategory	n^a^	Anchor example
Technical challenges	Data security	25	Violating patient and data privacy (Survey 2)
	Usability	17	Difficulties in usage (Survey 8)
	Software and hardware	12	Development of good software (Survey 26)
Costs and financing	Direct costs	34	Financing (Survey 138)
	Indirect costs	20	Time investment (Survey 48)
Therapeutic relationship and quality of therapy	Relationship	11	Difficulty relationship building (Survey 37)
	Quality of therapy	9	Tendency toward superficiality (Survey 19)
Adaptability and flexibility	Choice of digitally delivered interventions	15	How do I know for example, if an app is good? (Survey 173)
	Individualization	8	All of therapy needs to be adaptable to the patient (Survey 9)
Motivation and acceptance	Patients	14	Skepticism for example amongst older patients (Survey 22)
	Mental health professionals	11	Acceptance amongst mental health professionals (Survey 183)
Training and knowledge	Training	8	Further training is necessary (Survey 32)
	Knowledge and familiarity	7	Too little knowledge about digitally delivered interventions (Survey 178)
Indication and suitability	Contraindication	10	Not during crises (Survey 176)
	Judging risk	5	Risk of missing signs of suicidality (Survey 5)

a n: number of participants with code.

**Table 5.** Wishes for the future regarding blended therapy (BT).

Category	n^a^	Anchor example
Costs being covered	16	Costs covered by insurance (Survey 100)
Easy access	16	More easily accessible and nationally available offers (Survey 62)
Easy integration into therapy	16	BT as a self-evident part of the psychotherapeutic treatment (Survey 7)
Use as an add-on	13	Only as a supplement to face-to-face therapy (Survey 75)
Knowledge provision	12	More education and knowledge about it (Survey 130)
Individual tailoring	12	Good options that can be adapted by both mental health professional and patient (Survey 35)
Specialized programs	10	Diagnosis-specific implementation (Survey 37)
Software development	9	Good programs. I have tried Velibra, which I find very good (Survey 31)
Flexibility in use	9	Therapeutic freedom (Survey 42)
Studies on efficacy	9	Studies on effectiveness of digitally delivered interventions (Survey 182)
Training	8	Practice-based training (Survey 8)
Secure use	5	Moderately and with mindfulness towards the protection of personality (Survey 19)
Increased acceptance	4	More willingness/acceptance from all stakeholders (Survey 81)
Support for access for mental health professionals	4	First I want to be able to test the programs myself (Survey 93)
Program evaluation	4	Evidence-based programs (Survey 121)
No desire for more BT	3	Not everything needs to go into the direction of digitalization (Survey 25)

a n: number of participants with code.

## Discussion

### Principal Findings

This study explored mental health professionals’ perceptions of and experiences with BT. The quantitative findings indicate that participants report little knowledge of BT. Attitude toward BT was somewhat positive, and the acceptance of BT was moderate, comparable to previous literature from German-speaking countries [21] but divergent from a survey conducted with mental health professionals in the Netherlands where perceptions were generally positive [28]. This points to different perceptions of BT depending on the country in question and potential differing experiences with BT in different countries (see also Topooco et al [18] for a survey on attitudes toward digital interventions examined in different European countries). In Switzerland specifically, BT is not routinely implemented yet, and several applications of BT are currently not reimbursed by basic health insurance models. This specific barrier has also been highlighted in an interview study with executive staff and leadership of different Swiss psychiatric institutions, where cost coverage was mentioned as an important aspect [23].  

In addition, during recruitment, several professional associations, training institutions, and clinics actively declined to distribute the survey, citing staff shortages, an overload of inquiries, or other individual reasons. These experiences during recruitment may themselves potentially be indicative of broader attitudes toward blended therapy. Specifically, limited time resources or competing institutional priorities might reflect not only organizational constraints but also a lower perceived relevance or priority of BT within some professional contexts. Conversely, the fact that a considerable number of institutions were willing to disseminate the survey may point to growing awareness and openness toward the topic. This recruitment pattern could therefore indirectly mirror varying levels of acceptance or interest in BT among institutions and professionals, a finding that warrants further exploration in future research.

While most participants in our study reported some prior experience with BT, participants rarely used BT in the past 4 weeks. Additionally, the results revealed significant differences in the utilization of various digitally delivered intervention formats for BT, with teletherapy (video) being the most frequently used. Regarding suitability for BT, our study found significant differences between digitally delivered intervention types. Moreover, in our study, BT was deemed suitable for Mood disorders and Neurotic, stress-related, and Somatoform disorders by most participants (more than 75%, n=152), but suitable for Schizophrenia and delusional disorders or Intellectual disabilities by less than 20% of the participants. This may again in part be related to a lack of knowledge on BT, as studies have shown that digitally delivered interventions can also be successful as add-ons to treatment as usual for patients with schizophrenia-spectrum disorders [29] and that BT can be feasible for severe mental health disorders [30]. Willingness to use BT differed significantly between different treatment points. Descriptively, participants gave the lowest ratings for digitally delivered interventions as a substitute for face-to-face sessions. Willingness to use BT differed significantly across settings, with the lowest acceptance reported for acute inpatient care. This finding contrasts studies conducted on BT in the acute patient setting that show that stakeholders in acute inpatient care consider BT a suitable and relevant treatment option [31].

The qualitative analysis highlighted both perceived advantages and disadvantages of BT. Participants felt that BT can offer benefits, with therapy factors such as flexibility, outsourcing elements, and efficiency being most common. This aligns with the findings from a pilot trial on BT in Swiss outpatient care, where work independent of place and time was mentioned as a positive aspect of BT by therapists [32]. In our survey, patient factors included increased self-efficacy and lowered barriers to therapy. Strengthened therapeutic relationships and mental health professional−related benefits like enhanced capacity and remote work options further highlighted its practicality and appeal. The disadvantages reported by participants included additional effort, concerns about interpersonal interactions such as interruptions for the therapeutic relationship, and challenges with indication.

Aspects concerning the therapeutic relationship were considered both an advantage and a disadvantage of BT by mental health professionals. Interestingly, research shows that a therapeutic relationship can be established in digitally delivered interventions [33-36] and has, for example, been rated higher in BT than in usual care for depression [37]. This highlights a discrepancy between a polarized perception of the therapeutic relationship in BT by mental health professionals and the findings from empirical data on the therapeutic relationship in BT.

Regarding challenges for BT implementation, perceived hurdles included technical issues such as data security alongside direct and indirect costs. For the future, mental health professionals desire cost coverage of BT, accessibility, and easy integration of digitally delivered interventions into therapy. It should be noted that some of the aspects mentioned regarding cost coverage may be very specific to the Swiss context, where digital mental health interventions are currently mostly not included in basic health insurance models for patients.

### Future Directions

Nationally representative surveys assessing mental health professionals’ perceptions and experiences with BT should be conducted. In addition, it would be of interest to compare patient and mental health professional perspectives of BT using survey-based assessments. Moreover, longitudinal assessments should be used to examine BT perception changes over time. Finally, one future direction that seems particularly clinically relevant is to find effective ways of increasing knowledge on BT among therapy providers. This can be achieved by advancing information on BT in psychotherapy training but also by increasing exposure to digital interventions.

### Strengths and Limitations

To the best of our knowledge, this study is the first to investigate the topic of BT in depth among psychotherapists and psychiatrists (in training) in Switzerland. Recruitment strategies were broad (institutions, professional associations, clinics) with the aim of including a broad range of participants. Along with general perceptions of BT, modality-specific information was gained. Moreover, quantitative and qualitative methods were combined to analyze the data. The study also has limitations. First, the survey is not a representative sample of all psychotherapists and psychiatrists in Switzerland. It may have been biased, as only mental health professionals interested in BT filled out the survey. In addition, the distribution of professional experience in our sample was skewed, with more than one third of the participants reporting over 15 years of work experience, while only a small proportion had little or no experience. This uneven representation of experience levels limits the generalizability of our findings. Furthermore, our sample included different groups (eg, professional group or being in training vs not or therapeutic orientations). As shown in our multimedia appendices, some groups differed with regard to, for example, the use of specific digital interventions for BT. Moreover, the findings for a Swiss convenience sample may not translate to the perception of BT in other countries where, for example, attitudes toward digitally delivered intervention are more positive. Third, only a very short definition of BT was provided at the beginning of the survey. Thus, the concepts of BT may have differed widely between participants. While we decided to include the combination of teletherapy (video) and face-to-face sessions in our definition of BT, other studies have taken a different approach. Some equate videotherapy more with face-to-face treatment. Moreover, blended treatment has also been described as the combination of digital intervention and videotherapy [38]. Finally, the reported analyses provide a predominantly descriptive picture of cross-sectional data.

### Conclusions

While BT offers an innovative treatment option for patients with mental health disorders, mental health professionals report little knowledge, a somewhat positive attitude, and moderate acceptance. Both advantages and disadvantages of BT as perceived by mental health professionals were detailed in this study. Future implementation may be aided by increasing knowledge on BT for mental health professionals and in the Swiss context specifically by improving cost coverage options.

## Supplementary material

10.2196/78079 Multimedia Appendix 1 Survey.

10.2196/78079 Multimedia Appendix 2 Research questions and domains.

10.2196/78079 Multimedia Appendix 3 Information on missing data.

10.2196/78079 Multimedia Appendix 4 Supplementary analyses.

## Abbreviations

BT: blended therapy
*ICD-10*: *International Statistical Classification of Diseases, Tenth Revision*
MD: mean difference
