# Supplementary material for: Blended Therapy From the Perspective of Mental Health Professionals in Routine Mental Health Care: Mixed Methods Analysis of Cross-Sectional Survey Data
Source: JMIR Ment Health. 2026 Jan 6;13:e78079. doi: 10.2196/78079 (PMC12774310; doi:10.2196/78079)
Supplement: Multimedia Appendix 1 [file mental-v13-e78079-s001.docx]

### **Survey on Blended Therapy (translated from German)** *Note.* *The German version of the survey can be requested from the corresponding author upon reasonable request.*

1. **Introduction displayed to all participants:**

Dear Participants,

Thank you for your willingness to participate in this survey, which is being conducted by the University of Bern (Department of Clinical Psychology and Psychotherapy). We would like to learn more about your opinion on Blended Therapy!

The survey takes approximately 15-20 minutes.

The survey is anonymous. You can abort the survey at any time without giving reasons – data already collected will continue to be used for research purposes (including potential publication of results as part of student theses and possibly in journals and at conferences). The study has been approved by the Ethics Commission of the Faculty of Human Sciences at the University of Bern.

If you have any questions, please feel free to contact: *study* *email provided*

**I agree to participate in the survey and consent to the processing of my data under the stated conditions. I also confirm that I am a psychotherapist or psychiatrist in Switzerland or in training for these professions in Switzerland.**

o Yes

o No, I do not agree and do not want to participate

1. **Demographics**

**What gender do you identify with?**

o Male

o Female

o Non-binary (divers)

**How old are you (in years)?**

**_______**

**What professional group do you belong to or what title do you hold?**

o Federally recognized psychotherapist (or equivalent title)

o In training to become a federally recognized psychotherapist

o Specialist in psychiatry and psychotherapy

o In training to become a specialist in psychiatry and psychotherapy

o Specialist in child and adolescent psychiatry and psychotherapy

o In training to become a specialist in child and adolescent psychiatry and psychotherapy

*Display This Question:
If "What professional group do you belong to?" = In training to become a federally recognized psychotherapist OR If "What professional group do you belong to?" = In training to become a specialist in psychiatry and psychotherapy OR If "What professional group do you belong to?" = In training to become a specialist in child and adolescent psychiatry and psychotherapy*

***If in training, in which year?***

*o First year*

*o Second year*

*o Third year*

*o Fourth year*

*o Fifth year*

*o Sixth year*

*o In training for more than 6 years*

**How many years of professional experience (psychotherapeutic practice with patients) do you have?**

o None

o Less than 1 year

o 1-5 years

o 6-10 years

o 11-15 years

o More than 15 years

**What is your therapeutic orientation? (Multiple answers possible)**

o Cognitive-behavioral therapy (cognitive, cognitive-behavioral)

o Depth-psychological or psychodynamic

o Psychoanalytic

o Systemic

o Humanistic

o Other: (please specify) __________________________________________________

**What is your current work setting?**

o Outpatient

o Partial inpatient / day clinic

o Inpatient

o Mixed (outpatient and inpatient)

o Mixed (outpatient and partial inpatient)

o Mixed (partial inpatient and inpatient)

o Currently not employed

## **Survey Questions on BT**

### **How much do you already know about the topic of Blended Therapy?**

o Nothing at all

o Fairly little

o A little

o Some

o Fairly much

o A great deal

**Briefly describe what you understand the term "Blended Therapy" to mean:**

**What is Blended Therapy? A short description**

Blended Therapy refers to the combination of face-to-face psychotherapy with digitally delivered interventions. The technical design of these interventions varies widely. Examples include web-based programs, apps, interventions via email or chat, as well as the use of newer technologies (e.g., virtual reality or augmented reality).

Face-to-face psychotherapy can be combined with digitally delivered interventions in various ways. Here are some examples:

- Digitally delivered interventions are specifically and with intent used before or after face-to-face psychotherapy (e.g., stepped care or aftercare).
- Digitally delivered interventions are used as an additional element during psychotherapy.
- Face-to-face sessions alternate with digitally delivered interventions during psychotherapy (e.g., using an digital module as a substitute for a face-to-face session).

What is NOT considered Blended Therapy?

The exclusive use of digitally delivered interventions (without any combination with face-to-face contact) is **NOT** considered Blended Therapy. For example, therapies that are conducted exclusively via video conferencing (screen-to-screen therapy) are not considered Blended Therapy. The use of digitally delivered interventions solely as administrative tools during face-to-face psychotherapy (e.g., scheduling appointments via WhatsApp) is also not considered Blended Therapy.

**What is your attitude towards Blended Therapy?**

o Strongly negative

o Negative

o Somewhat negative

o Neutral

o Somewhat positive

o Positive

o Strongly positive

**How suitable do you consider the combination of face-to-face psychotherapy with the following digitally delivered interventions for the treatment of people with mental health disorders?**

| Intervention Type | Not Suitable | Rather Not Suitable | Unclear | Rather Suitable | Suitable |
| --- | --- | --- | --- | --- | --- |
| Teletherapy (video) | o | o | o | o | o |
| Intervention via chat (real-time) | o | o | o | o | o |
| Intervention via email | o | o | o | o | o |
| Self-management intervention (e.g., web-based program, platform, or app) | o | o | o | o | o |
| New technologies (e.g., virtual reality, augmented reality) | o | o | o | o | o |

**For which diagnostic categories (ICD-10) do you consider Blended Therapy to be suitable? (Multiple choices possible)**

| Diagnostic Category | Yes, Suitable | If yes, why? | |
| --- | --- | --- | --- |
| F00-F09 Organic, including symptomatic mental disorders | o |  |  |
| F10-F19 Mental and behavioral disorders due to psychoactive substance use | o |  |  |
| F20-F29 Schizophrenia, schizotypal, and delusional disorders | o |  |  |
| F30-F39 Affective disorders | o |  |  |
| F40-F48 Neurotic, stress-related, and somatoform disorders | o |  |  |
| F50-F59 Behavioral syndromes associated with physiological disturbances and physical factors | o |  |  |
| F60-F69 Personality and behavioral disorders | o |  |  |
| F70-F79 Intellectual disabilities | o |  |  |
| F80-F89 Developmental disorders | o |  |  |
| F90-F98 Behavioral and emotional disorders with onset usually occurring in childhood and adolescence | o |  |  |

*This following question is only presented when participants stated yes in the previous question. The same question format is presented for all other disorder categories ranging from F0-F9:*

**How suitable do you consider the combination of face-to-face psychotherapy with the following digitally delivered interventions for the treatment of Mental and behavioral disorders due to psychoactive substance use (F10-19)?**

| Intervention Type | Not Suitable | Rather Not Suitable | Unclear | Rather Suitable | Suitable |
| --- | --- | --- | --- | --- | --- |
| Teletherapy (video) | o | o | o | o | o |
| Intervention via chat (real-time) | o | o | o | o | o |
| Intervention via email | o | o | o | o | o |
| Self-management intervention (e.g., web-based program, platform, or app) | o | o | o | o | o |
| New technologies (e.g., Virtual Reality, Augmented Reality) | o | o | o | o | o |

**Are there specific treatment situations where Blended Therapy is particularly suitable?**

**_______**_________________________________________________________________________________________

**I could imagine including Blended Therapy into my work.**

o Totally disagree

o Rather disagree

o Neutral (*weder noch*)

o Rather agree

o Totally agree

**I intend to try Blended Therapy in my work within the next year.**

o Totally disagree

o Rather disagree

o Neutral (*weder noch*)

o Rather agree

o Totally agree

**How high is your intention to use Blended Therapy in your work ever?**

0% (No intention) → 100% (Full intention) *- displayed as a slider*

**Does the face-to-face part of psychotherapy change due to the combination with an digitally delivered intervention?**

o Yes

o No

o I don’t know

(If "Yes," the following question is displayed.)

**How does the use of an digitally delivered intervention potentially change the face-to-face part of psychotherapy, and which aspects of it?**

**Did you offer any form of Blended Therapy in the past four weeks?**

o Not at all

o Rarely

o Occasionally

o Often

o Very often

*If "Rarely" to "Very often” was picked in the previous question, the following question is displayed:*

**Which of the following digitally delivered interventions have you combined with face-to-face psychotherapy in the past four weeks? (Multiple selections possible)**

o Teletherapy (video) (yes/no)

o Intervention via chat (real-time) (yes/no)

o Intervention via email (yes/no)

o Self-management intervention (e.g., web-based program, platform, or app) (yes/no)

o New technologies (e.g., virtual reality or augmented reality) (yes/no)

**Have you ever offered Blended Therapy in the past (more than one month ago)?**

o Yes

o No

(If "Yes," the following question is displayed.)

**Which of the following digitally delivered interventions have you combined with face-to-face psychotherapy in the past? (Multiple selections possible)**

o Teletherapy (video)

o Intervention via chat (real-time)

o Intervention via email

o Self-management intervention (e.g., web-based program, platform, or app)

o New technologies (e.g., virtual reality or augmented reality)

**What are the potential advantages for you if you provide or increase providing Blended Therapy in the future?**

**What are the potential disadvantages for you if you provide or increase providing Blended Therapy in the future?**

**Would you be willing to offer the following Blended Therapy options in the future for patients?**

| Blended Therapy Option | Definitely No | Rather No | Rather Yes | Definitely Yes |
| --- | --- | --- | --- | --- |
| Digitally delivered interventions before therapy (e.g., during wait times for a therapy slot) | o | o | o | o |
| Digitally delivered interventions after therapy (e.g., follow-up care) | o | o | o | o |
| Digitally delivered interventions during therapy | o | o | o | o |
| Digitally delivered interventions replacing individual face-to-face sessions | o | o | o | o |
| Digitally delivered interventions replacing parts within a face-to-face session | o | o | o | o |

**Would you be willing to offer the following Blended Therapy options for patients in the future?**

| Blended Therapy Option | Definitely No | Rather No | Rather Yes | Definitely Yes |
| --- | --- | --- | --- | --- |
| Digitally delivered interventions that are strongly integrated with face-to-face sessions (high connection between face-to-face sessions and DIGITALLY DELIVERED INTERVENTION) | o | o | o | o |
| Digitally delivered interventions that are loosely integrated with face-to-face sessions (low connection between face-to-face sessions and DIGITALLY DELIVERED INTERVENTION) | o | o | o | o |

**Would you be willing to offer Blended Therapy options in the following settings in the future?**

| Setting | Definitely No | Rather No | Rather Yes | Definitely Yes |
| --- | --- | --- | --- | --- |
| Acute inpatient setting | o | o | o | o |
| Inpatient setting | o | o | o | o |
| Day clinic setting | o | o | o | o |
| Outpatient setting | o | o | o | o |

**Would you be willing to offer the following Blended Therapy options for patients in the future?**

| Blended Therapy Option | Definitely No | Rather No | Rather Yes | Definitely Yes |
| --- | --- | --- | --- | --- |
| Blended Therapy with a structured guideline on how exactly a digitally delivered intervention and face-to-face psychotherapy should be combined (predefined procedure) | o | o | o | o |
| Blended Therapy where patients and/or therapists design the procedure themselves | o | o | o | o |

**Which elements of psychotherapy would you like to offer via a digitally delivered intervention in the future?**

**What proportion of digitally delivered interventions and face-to-face sessions do you find ideal for psychotherapy?**

Percentage of psychotherapy that takes place face-to-face:
0% = No face-to-face contact at all → 100%= – entire therapy face-to-face *(presented as a slider)*

**What type of funding would you prefer for Blended Therapy in Switzerland?**

o If costs arise for digitally delivered interventions in combination with psychotherapy, patients should cover costs themselves (self-payment).

o Health insurance companies should cover the cost of Digitally delivered interventions in combination with face-to-face psychotherapy.

o Digitally delivered interventions combined with face-to-face psychotherapy should be funded by the government.

**What are your wishes for the future regarding Blended Therapy?**

**What are the challenges regarding the implementation of Blended Therapy?**

**What type of support would be useful for you to offer or expand offering Blended Therapy in the future? (Multiple choices possible)**

o Support through courses in basic training (university studies)

o Support through courses in further training

o Support through self-study materials (guidelines, directories of Digitally delivered interventions)

o Supervision at the workplace

o IT support at the workplace

o Provision of Digitally delivered interventions

o Courses on Blended Therapy at the workplace

o No support needed

o Other (please specify): _______
