# Supplementary material for: Blended Therapy From the Perspective of Mental Health Professionals in Routine Mental Health Care: Mixed Methods Analysis of Cross-Sectional Survey Data
Source: JMIR Ment Health. 2026 Jan 6;13:e78079. doi: 10.2196/78079 (PMC12774310; doi:10.2196/78079)
Supplement: Multimedia Appendix 2 [file mental-v13-e78079-s002.docx]

**Research questions and domains mapped to survey items**

Survey items and their response formats as related to the research questions and domains of the research questions.

| **Research Question - Domain** | **Item** | **Response format / scale** |
| --- | --- | --- |
| **RQ 1- Perceived knowledge** | How much do you already know about the topic of Blended Therapy? | 1 = Nothing at all 2 = Fairly little 3 = A little  4 = Some 5 = Fairly much 6 = A great deal |
| **RQ 1 - Attitude** | What is your attitude towards Blended Therapy? | 1 = Strongly negative 2 = Negative  3 = Somewhat negative 4 = Neutral  5 = Somewhat positive  6 = Positive  7 = Strongly positive |
| **RQ 1 - Imagine** | I could imagine including Blended Therapy into my work. | 1 = Totally disagree 2 = Rather disagree  3 = Neutral *(weder noch)*  4 = Rather agree  5 = Totally agree |
| **RQ 1 - Intend to try** | I intend to try Blended Therapy in my work within the next year | 1 = Totally disagree 2 = Rather disagree  3 = Neutral *(weder noch)*  4 = Rather agree  5 = Totally agree |
| **RQ 1 - Intention to use** | How high is your intention to use Blended Therapy in your work ever? | 0 % (no intention at all) - 100% 1-5 Likert scale converted |
| **RQ 1 - Acceptance** | *Acceptance score across Imagine, Intend to try, Intention to use* | *Composite (mean of 3 items)* |
| **RQ 2 - Current use** | Did you offer any form of Blended Therapy in the past four weeks? | 1 = Not at all  2 = Rarely  3 = Occasionally  4 = Often  5 = Very often |
| **RQ 2 - Past use** | Have you ever offered Blended Therapy in the past (more than one month ago)? | Yes / No |
| **RQ 2 - Type of intervention currently used in BT** | Which of the following digitally delivered interventions have you combined with face-to-face psychotherapy in the past four weeks? (Multiple selections possible): Teletherapy (video) | Yes /No |
| **RQ 2 - Type of intervention currently used in BT** | Which of the following digitally delivered interventions have you combined with face-to-face psychotherapy in the past four weeks? (Multiple selections possible): Intervention via chat (real-time) | Yes / No |
| **RQ 2 - Type of intervention currently used in BT** | Which of the following digitally delivered interventions have you combined with face-to-face psychotherapy in the past four weeks? (Multiple selections possible): Intervention via email | Yes / No |
| **RQ 2 - Type of intervention currently used in BT** | Which of the following digitally delivered interventions have you combined with face-to-face psychotherapy in the past four weeks? (Multiple selections possible): Self-management intervention (e.g., web-based program, platform, or app) | Yes / No |
| **RQ 2 - Type of intervention currently used in BT** | Which of the following digitally delivered interventions have you combined with face-to-face psychotherapy in the past four weeks? (Multiple selections possible): New technologies (e.g., virtual reality or augmented reality) | Yes / No |
| **RQ3 – Suitability of digital interventions** | How suitable do you consider the combination of face-to-face psychotherapy with the following digitally delivered interventions for the treatment of people with mental health disorders?: Teletherapy (video) | 1 = Not suitable  2 = Rather not suitable  3 = Unclear  4 = Rather suitable  5 = Suitable |
| **RQ3 – Suitability of digital interventions** | How suitable do you consider the combination of face-to-face psychotherapy with the following digitally delivered interventions for the treatment of people with mental health disorders?: Intervention via chat (real-time) | 1 = Not suitable  2 = Rather not suitable  3 = Unclear  4 = Rather suitable  5 = Suitable |
| **RQ3 – Suitability of digital interventions** | How suitable do you consider the combination of face-to-face psychotherapy with the following digitally delivered interventions for the treatment of people with mental health disorders?: Intervention via email | 1 = Not suitable  2 = Rather not suitable  3 = Unclear  4 = Rather suitable  5 = Suitable |
| **RQ3 – Suitability of digital interventions** | How suitable do you consider the combination of face-to-face psychotherapy with the following digitally delivered interventions for the treatment of people with mental health disorders?: Self-management intervention (e.g., web-based program, platform, or app) | 1 = Not suitable  2 = Rather not suitable  3 = Unclear  4 = Rather suitable  5 = Suitable |
| **RQ3 – Suitability of digital interventions** | How suitable do you consider the combination of face-to-face psychotherapy with the following digitally delivered interventions for the treatment of people with mental health disorders?: New technologies (e.g., Virtual Reality, Augmented Reality) | 1 = Not suitable  2 = Rather not suitable  3 = Unclear  4 = Rather suitable  5 = Suitable |
| **RQ3 – Suitability for ICD-10 disorders** | For which diagnostic categories (ICD-10) do you consider Blended Therapy to be suitable? (Multiple choices of ICD-10 categories possible) | Yes, suitable (choice) |
| **RQ3 – Willingness (treatment phase)** | Would you be willing to offer the following Blended Therapy options in the future for patients? Digitally delivered interventions before therapy (e.g., during wait times for a therapy slot) | 1 = Definitely no  2 = Rather no  3 = Rather yes  4 = Definitely yes |
| **RQ3 – Willingness (treatment phase)** | Would you be willing to offer the following Blended Therapy options in the future for patients? Digitally delivered interventions after therapy (e.g., follow-up care) | 1 = Definitely no  2 = Rather no  3 = Rather yes  4 = Definitely yes |
| **RQ3 – Willingness (treatment phase)** | Would you be willing to offer the following Blended Therapy options in the future for patients? Digitally delivered interventions during therapy | 1 = Definitely no  2 = Rather no  3 = Rather yes  4 = Definitely yes |
| **RQ3 – Willingness (treatment phase)** | Would you be willing to offer the following Blended Therapy options in the future for patients? Digitally delivered interventions replacing individual face-to-face sessions | 1 = Definitely no  2 = Rather no  3 = Rather yes  4 = Definitely yes |
| **RQ3 – Willingness (treatment phase)** | Would you be willing to offer the following Blended Therapy options in the future for patients? Digitally delivered interventions replacing parts within a face-to-face session | 1 = Definitely no  2 = Rather no  3 = Rather yes  4 = Definitely yes |
| **RQ3 – Willingness (setting)** | Would you be willing to offer Blended Therapy options in the following settings in the future? Acute inpatient setting | 1 = Definitely no  2 = Rather no  3 = Rather yes  4 = Definitely yes |
| **RQ3 – Willingness (setting)** | Would you be willing to offer Blended Therapy options in the following settings in the future? Inpatient setting | 1 = Definitely no  2 = Rather no  3 = Rather yes  4 = Definitely yes |
| **RQ3 – Willingness (setting)** | Would you be willing to offer Blended Therapy options in the following settings in the future? Day clinic setting | 1 = Definitely no  2 = Rather no  3 = Rather yes  4 = Definitely yes |
| **RQ3 – Willingness (setting)** | Would you be willing to offer Blended Therapy options in the following settings in the future? Outpatient setting | 1 = Definitely no  2 = Rather no  3 = Rather yes  4 = Definitely yes |
| **RQ4 - advantages, disadvantages, challenges and wishes for the future (open-ended questions)** | What are the potential advantages for you if you provide or increase providing BT in the future?  What are the potential disadvantages for you if you provide or increase providing BT in the future?  What are the challenges regarding the implementation of BT?  What are your wishes for the future regarding BT? | |

*Notes*. *BT* = blended therapy; *SD* = standard deviation; *RQ* = research question
