# Supplementary material for: Blended Therapy From the Perspective of Mental Health Professionals in Routine Mental Health Care: Mixed Methods Analysis of Cross-Sectional Survey Data
Source: JMIR Ment Health. 2026 Jan 6;13:e78079. doi: 10.2196/78079 (PMC12774310; doi:10.2196/78079)
Supplement: Multimedia Appendix 3 [file mental-v13-e78079-s003.docx]

**Details on missing data**

Demographic items, items regarding prior knowledge and attitudes toward BT, the assessment of the suitability of different digitally delivered intervention dimensions, and the intention to use BT, were answered by all 203 participants. Likewise, all respondents indicated whether they are currently or have in the past offered some form of BT. Concerning specific willingness for future applications of BT, 198 participants (97.5%) responded to the item of whether digitally delivered interventions should be used before therapy, and 199 (98%) answered questions about the use of digitally delivered interventions after therapy, during psychotherapy, or as a replacement for individual face-to-face therapy sessions. For the item asking whether digitally delivered interventions would be used in the future as a substitute for certain parts of a face-to-face session, 193 (95.1%) provided a response. The assessment of the suitability of BT in different settings (four items) showed a greater variability in response rates. 196 (96.6%) participants assessed suitability in the outpatient setting, 180 (88.7%) for the acute inpatient setting, 176 (86.7%) for the day clinic setting, and 175 (86.2%) provided an assessment for the inpatient setting. For the open-ended questions that were analyzed qualitatively, 148 (72.9%) participants responded to the question about advantages of BT, 141 (69.5%) participants reported on disadvantages of BT, a total of 129 (63.5%) individuals were included in the qualitative analysis of open-ended responses to the item on challenges of BT implementation and 108 (53.2%) reported on wishes for future implementation.
