# Supplementary material for: Blended Therapy From the Perspective of Mental Health Professionals in Routine Mental Health Care: Mixed Methods Analysis of Cross-Sectional Survey Data
Source: JMIR Ment Health. 2026 Jan 6;13:e78079. doi: 10.2196/78079 (PMC12774310; doi:10.2196/78079)
Supplement: Multimedia Appendix 4 [file mental-v13-e78079-s004.docx]

### **Supplementary analyses**

**Table S1.** Counts and percentages for the item on knowledge of BT.

|  | Nothing at all | Fairly little | A little | Some | Fairly much | A great deal | |
| --- | --- | --- | --- | --- | --- | --- | --- |
| N (%) | 44 (21.7) | 50 (24.6) | 55 (27.1) | 32 (15.8) | 18 (8.9) | | 4 (2.0) |

**Note.** N = 203. Item: How much do you already know about the topic of Blended Therapy?

**Table S2.** Perceived knowledge of BT by professional group and being in training.

| **Group** | **n** | **Mean (SD)** | **t (df)** | **P** |
| --- | --- | --- | --- | --- |
| **Professional group** |  |  |  |  |
| Psychologists | 152 | 2.76 (1.35) | — | — |
| Psychiatrists | 51 | 2.59 (1.20) | 0.79 (201) | .43 |
| **Training group** |  |  |  |  |
| In training | 68 | 2.63 (1.40) | — | — |
| Specialist title | 135 | 2.76 (1.28) | −0.63 (201) | .53 |

**Note.** N = 203. M = Mean, SD = Standard deviation. Nothing at all = 1, a little = 3, a great deal = 6.

**Table S3.** Attitude toward BT by professional group and being in training.

| **Group** | **n** | **Mean (SD)** | **t (df)** | **P** |
| --- | --- | --- | --- | --- |
| **Professional group** |  |  |  |  |
| Psychologists | 152 | 5.29 | — | — |
| Psychiatrists | 51 | 5.14 | .72 (201) | 0.48 |
| **Training group** |  |  |  |  |
| In training | 68 | 5.49 | — | — |
| Specialist title | 135 | 5.13 | 1.77(201) | 0.08 |

**Note.** N = 203. M = Mean, SD = Standard deviation. Strongly negative = 1, neutral = 4, Strongly positive = 7.

**Table S4.** Mean values for the acceptance of BT by professional group and being in training.

| **Group** | **n** | **Mean (SD)** | **t (df)** | **P** |
| --- | --- | --- | --- | --- |
| **Total sample** | 203 | 3.64 (1.20) | — | — |
| **Professional group** |  |  |  |  |
| Psychologists | 152 | 3.64 (1.18) | — | — |
| Psychiatrists | 51 | 3.63 (1.25) | 0.06 (201) | .96 |
| **Training status** |  |  |  |  |
| In training | 68 | 3.57 (1.10) | — | — |
| Specialist title | 135 | 3.67 (1.25) | −0.52 (201) | .60 |

**Note.** N = 203. M = Mean, SD = Standard deviation

**Table S5.** Pairwise McNemar tests for current use of BT for different digitally delivered intervention formats (N = 203)**.**

| **Digitally delivered intervention comparison** | **χ² (df =1)** | **P** | | **Bonferroni-corrected p*(10 comparisons)** | **Cohen’s g** | **Magnitude**^a^ |
| --- | --- | --- | --- | --- | --- | --- |
| Video vs Chat | 51.19 | | < .001 | < .001 | 0.44 | large |
| Video vs Email | 6.78 | | .009 | .09 | 0.18 | medium |
| Video vs Self-management | 21.55 | | < .001 | < .001 | 0.27 | large |
| Video vs New technology | 79.01 | | < .001 | < .001 | 0.50 | large |
| Chat vs Email | 24.48 | | < .001 | < .001 | 0.33 | large |
| Chat vs Self-management | 5.35 | | .02 | .21 | 0.17 | medium |
| Chat vs New technology | ^b^— | | ^b^— | .000 | 0.42 | large |
| Email vs Self-management | 6.35 | | .01 | .12 | 0.20 | medium |
| Email vs New technology | 54.39 | | < .001 | < .001 | 0.47 | large |
| Self-management vs New technology | 35.22 | | < .001 | < .001 | 0.47 | large |

**Note.** Bonferroni correction applied for 10 pairwise comparisons (adjusted α = .005). ^a^Magnitude interpretation: small = 0.10, medium = 0.30, large = 0.50 (adapted from Cohen). ^b^For Chat vs. new technology counts were low, so SPSS provided the exact significance test only, using binomial distribution.

**Table S6**. Current and past use of blended therapy by professional group and being in training.

| **Group** | **n** | **Past use, n (%)** | **Current use, mean (SD)** |
| --- | --- | --- | --- |
| **Total sample** | 203 | 125 (61.6) | 2.14 (1.22) |
| **Professional group** |  |  |  |
| Psychologists | 152 | 96 (63.2) | 2.16 (1.26) |
| Psychiatrists | 51 | 29 (56.9) | 2.06 (1.09) |
| t (df) |  |  | 0.54 (201) |
| P |  |  | .59 |
| **Training status** |  |  |  |
| In training | 68 | 35 (51.5) | 1.90 (1.16) |
| Specialist title | 135 | 90 (66.7) | 2.26 (1.23) |
| t (df) |  |  | -2.02 (201) |
| P |  |  | .05 |

**Note.** N = 203. M = Mean; SD = Standard deviation. Past use = Yes or No. Current use = 1 (*not at all*) to 5 (*very often*).

**Table S7a.** Current use of digitally delivered interventions according to professional groups.

| **Digital modality** | **Total sample, n (%)** | **Psychologists, n (%)** | **Psychiatrists, n (%)** | **χ² (df =1)** | **P** | **Padj** |
| --- | --- | --- | --- | --- | --- | --- |
| Teletherapy (video) | 87 (42.9) | 69 (45.39) | 18 (35.29) | 1.59 | .21 | 1.00 |
| Chat | 27 (13.3) | 21 (13.82) | 6 (11.76) | 0.14 | .71 | 1.00 |
| E-mail | 66 (32.5) | 45 (29.61) | 21 (41.18) | 2.33 | .13 | 1.00 |
| Self-management intervention | 45 (22.2) | 34 (22.37) | 11 (21.57) | 0.01 | .91 | 1.00 |
| New technologies | 6 (3.0) | 6 (3.95) | 0 (0) | 2.07 | .15 | 1.00 |

**Note.** N = 203. χ² tests compared the current use of each digital modality between professional groups (psychologists vs psychiatrists). Padj values reflect Bonferroni-corrected P values.

**Table S7b.** Current use of digitally delivered interventions according to in training vs. not

| **Digital modality** | **In training, n (%)** | **Licensed therapist, n (%)** | **χ² (1)** | **P** | **Padj** |
| --- | --- | --- | --- | --- | --- |
| Teletherapy (video) | 19 (27.90) | 68 (50.4) | 9.29 | .002 | .02 |
| Chat | 5 (7.4) | 22 (16.3) | 3.14 | .08 | .80 |
| E-mail | 18 (26.5) | 48 (35.6) | 1.70 | .19 | 1.00 |
| Self-management interventions | 17 (25.0) | 28 (20.7) | 0.48 | .49 | 1.00 |
| New technologies | 3 (4.4) | 3 (2.2) | 0.76 | .39 | 1.00 |

**Note.** N = 203. χ² test was conducted to compare current use of different digital modalities between different professional groups and between those in training vs. not in training. Padj values reflect Bonferroni-corrected P values.

**Table S8.** Descriptive values for suitability for digitally delivered intervention dimensions

|  | Video conference (M;SD) | Intervention via chat^a^ (M;SD) | Intervention by e-mail (M;SD) | Self-management^b^ (M;SD) | New technologies^c^ (M;SD) |
| --- | --- | --- | --- | --- | --- |
| Total sample (N = 203) | 4.05 (.98) | 3.27 (1.24) | 3.20 (1.23) | 3.80 (1.07) | 3.52 (1.07) |

**Note.** ^b^ e.g. web-based program, platform or app ^c^ e.g. Virtual Reality, Augmented Reality

**Table S9.** Pairwise McNemar tests for general suitability of BT for different ICD-10 categories

| **ICD-10 category comparison** | **χ² (continuity adjusted)** | **P** | **Bonferroni-corrected P** | **Cohen’s g** | **Magnitude** |
| --- | --- | --- | --- | --- | --- |
| F30–F39 vs F80–F89 | 117.60 | < .001 | < .001 | 0.47 | Large |
| F30–F39 vs F70–F79 | 134.06 | < .001 | < .001 | 0.49 | Large |
| F30–F39 vs F40–F48 | 4.97 | .03 | 1.00 | 0.21 | Large |
| F30–F39 vs F60–F69 | 47.78 | < .001 | < .001 | 0.43 | Large |
| F30–F39 vs F10–F19 | 40.98 | < .001 | < .001 | 0.42 | Large |
| F30–F39 vs F20–F29 | 133.01 | < .001 | < .001 | 0.50 | Large |
| F30–F39 vs F90–F98 | 76.86 | < .001 | < .001 | 0.47 | Large |
| F30–F39 vs F50–F59 | 73.29 | < .001 | < .001 | 0.32 | Large |
| F30–F39 vs F00–F09 | 112.62 | < .001 | < .001 | 0.49 | Large |
| F80–F89 vs F70–F79 | 3.70 | .05 | 1.00 | 0.20 | Medium |
| F80–F89 vs F40–F48 | 101.98 | < .001 | < .001 | 0.47 | Large |
| F80–F89 vs F60–F69 | 50.81 | < .001 | < .001 | 0.39 | Large |
| F80–F89 vs F10–F19 | 62.50 | < .001 | < .001 | 0.35 | Large |
| F80–F89 vs F20–F29 | 1.02 | .31 | 1.00 | 0.13 | Small–medium |
| F80–F89 vs F90–F98 | 29.09 | < .001 | < .001 | 0.42 | Large |
| F80–F89 vs F50–F59 | 30.95 | < .001 | < .001 | 0.44 | Large |
| F80–F89 vs F00–F09 | 0.37 | .54 | 1.00 | 0.17 | Medium |
| F70–F79 vs F40–F48 | 112.90 | < .001 | < .001 | 0.49 | Large |
| F70–F79 vs F60–F69 | 67.84 | < .001 | < .001 | 0.43 | Large |
| F70–F79 vs F10–F19 | 76.25 | < .001 | < .001 | 0.42 | Large |
| F70–F79 vs F20–F29 | 0.10 | .75 | 1.00 | 0.08 | Small |
| F70–F79 vs F90–F98 | 37.16 | < .001 | < .001 | 0.35 | Large |
| F70–F79 vs F50–F59 | 42.56 | < .001 | < .001 | 0.35 | Large |
| F70–F79 vs F00–F09 | 5.36 | .02 | .95 | 0.24 | Large |
| F40–F48 vs F60–F69 | 28.02 | < .001 | < .001 | 0.40 | Large |
| F40–F48 vs F10–F19 | 17.75 | < .001 | < .001 | 0.43 | Large |
| F40–F48 vs F20–F29 | 115.20 | < .001 | < .001 | 0.49 | Large |
| F40–F48 vs F90–F98 | 56.01 | < .001 | < .001 | 0.44 | Large |
| F40–F48 vs F50–F59 | 61.04 | < .001 | < .001 | 0.49 | Large |
| F40–F48 vs F00–F09 | 98.70 | < .001 | < .001 | 0.57 | Large |
| F60–F69 vs F10–F19 | 0.51 | .48 | 1.00 | 0.10 | Small |
| F60–F69 vs F20–F29 | 67.95 | < .001 | < .001 | 0.50 | Large |
| F60–F69 vs F90–F98 | 9.35 | .002 | .09 | 0.21 | Medium |
| F60–F69 vs F50–F59 | 9.77 | .002 | .09 | 0.22 | Medium |
| F60–F69 vs F00–F09 | 45.10 | < .001 | < .001 | 0.45 | Large |
| F10–F19 vs F20–F29 | 73.29 | < .001 | < .001 | 0.32 | Large |
| F10–F19 vs F90–F98 | 16.75 | < .001 | < .001 | 0.30 | Large |
| F10–F19 vs F50–F59 | 12.64 | < .001 | < .001 | 0.25 | Large |
| F10–F19 vs F00–F09 | 52.69 | < .001 | < .001 | 0.36 | Large |
| F20–F29 vs F90–F98 | 34.39 | < .001 | < .001 | 0.40 | Large |
| F20–F29 vs F50–F59 | 35.21 | < .001 | < .001 | 0.40 | Large |
| F20–F29 vs F00–F09 | 2.72 | .10 | 1.00 | 0.27 | Large |
| F90–F98 vs F50–F59 | 0.02 | .90 | 1.00 | 0.03 | Small |
| F90–F98 vs F00–F09 | 17.50 | < .001 | < .001 | 0.41 | Large |
| F50–F59 vs F00–F09 | 21.39 | < .001 | < .001 | 0.42 | Large |

**Note.** χ² = chi-square test statistic (continuity adjusted). Bonferroni correction was applied for multiple pairwise comparisons. Cohen’s g indicates effect size; magnitudes were interpreted as small = 0.05–0.15, medium = 0.15–0.25, and large > 0.25.
